# Supplementary figures and images for: The Drosophila Baramicin polypeptide gene protects against fungal infection
Source: PLoS Pathog. 2021 Aug 25;17(8):e1009846. doi: 10.1371/journal.ppat.1009846 (PMC8423362; doi:10.1371/journal.ppat.1009846)

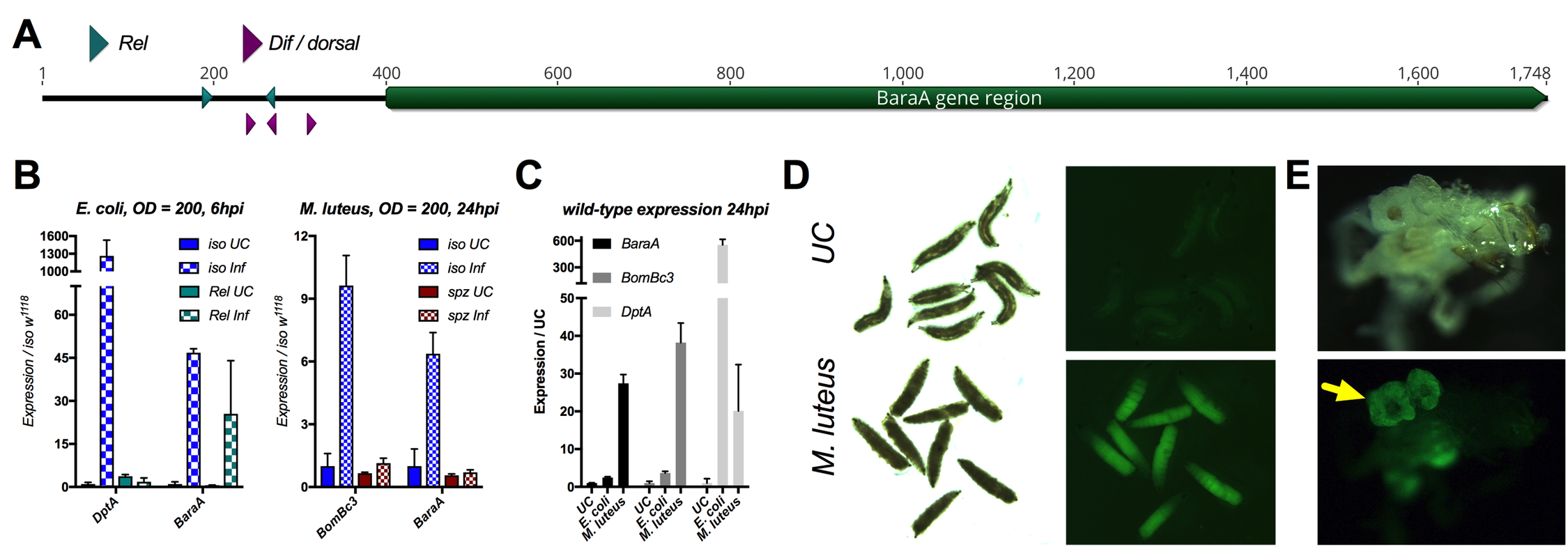

Supplement: S1 Fig — A) 400bp of upstream sequence from BaraA annotated with putative Rel or Dif/dl binding sites (included in S1 Data). B) Expression of BaraA in wild-type and spzrm7 flies following injury with the Gram-negative bacterium E. coli or the Gram-positive bacterium M. luteus. As seen in a previous microarray (Fig 1A), basal BaraA expression is depressed in RelE20 flies, but is nevertheless highly induced upon infection, likely representing the BaraA response to injury. C) In a separate set of experiments, BaraA returns to near-baseline levels of expression by 24hpi using E. coli. Meanwhile BaraA remained induced after pricking with M. luteus, mirroring the Toll-regulated BomBc3 but not the Imd-regulated DptA. D) The BaraA>mGFP reporter line shows a robust induction of GFP 2hpi upon pricking with M. luteus in larvae. E) Expression of BaraA>mGFP in the spermatheca of females (yellow arrow). Representative images shown. (TIF) [file ppat.1009846.s001.tif]

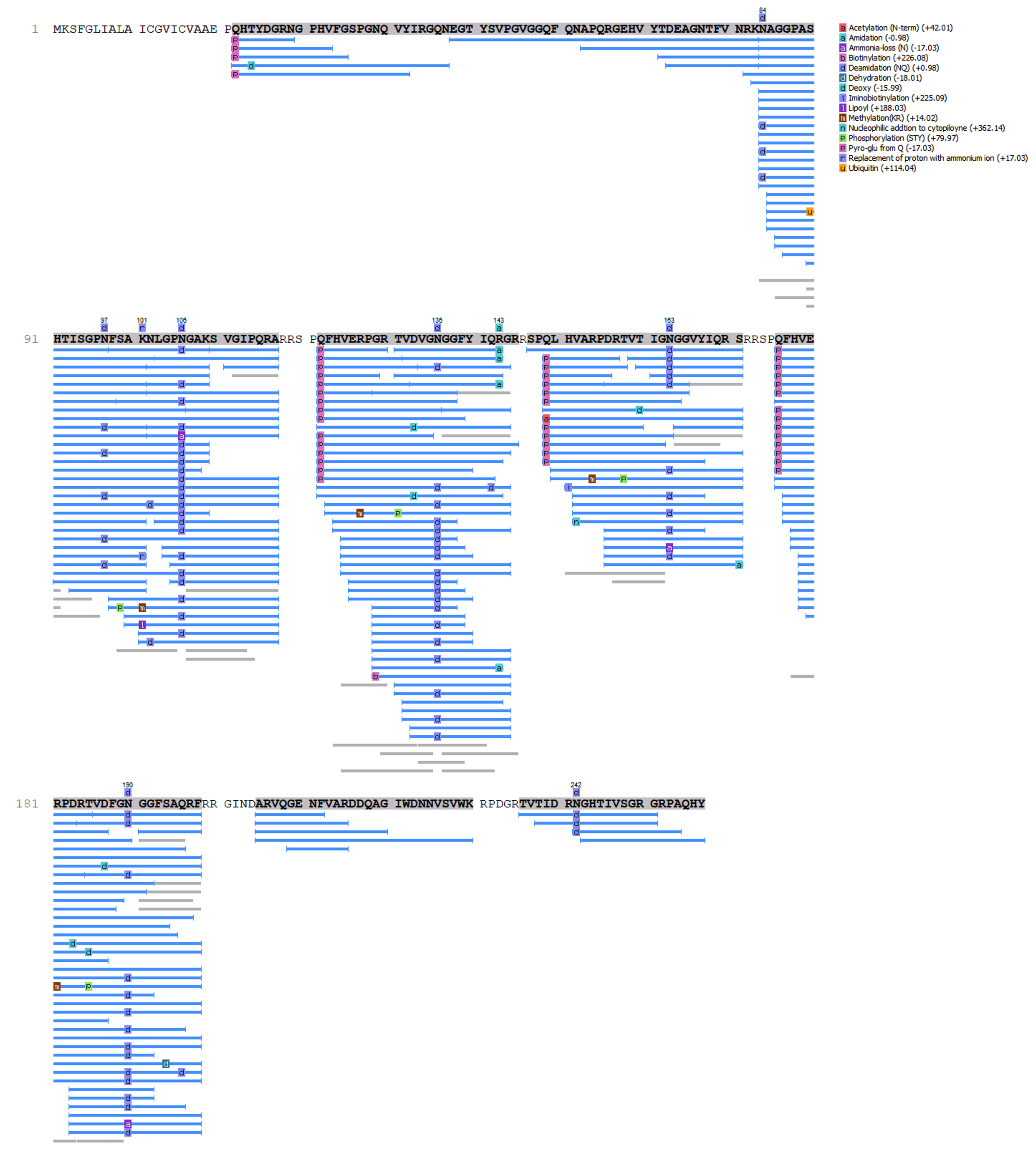

Supplement: S2 Fig — Detected peptide fragments (blue bars) cover the whole precursor protein barring furin site-associated motifs. Additionally, two peptide fragments are absent: i) the first 4 residues of the C-terminus (“GIND,” not predicted a priori), and ii) the C-terminus peptide’s “RPDGR” motif, which is predicted as a degradation product of Trypsin cleavage and whose size is beyond the minimum range of detection. Without the GIND motif, the mass of the contiguous C-terminus is 5974.5 Da, matching the mass observed by MALDI-TOF for IM22 (Fig 2A). The N-terminal Q residues of IM10, IM12, IM13, and IM24 are pyroglutamate-modified, as described previously [24]. The asparagine residues of IM10-like peptides are sometimes deamidated, likely as a consequence of our 0.1% TFA sample collection method as “NG” motifs are deamidated in acidic conditions [58]. (TIF) [file ppat.1009846.s002.tif]

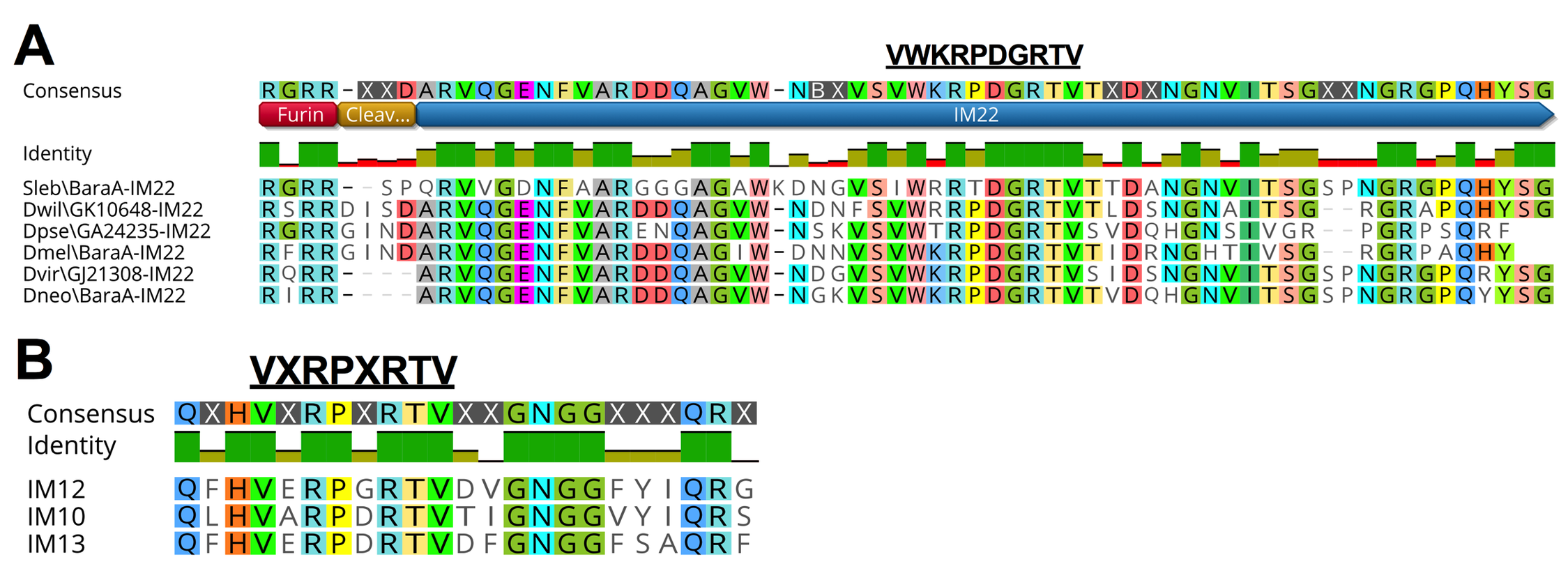

Supplement: S3 Fig — A) Aligned IM22 peptides of Drosophila Baramicin A-like genes, with the IM10-like ‘VWKRPDGRTV’ motif noted. The GIND residues at the N-terminus are cleaved off in Dmel\BaraA by an unknown process, and this subsequent peptide is similarly cleaved following RXRR furin cleavage sites in subgenus Drosophila flies. As a consequence, the mature IM22 peptide is predicted to be the same across species even when different cleavage mechanisms are utilized. B) Alignment of the three IM10-like peptides of D. melanogaster BaraA with the “VXRPXRTV” motif noted. The residue 8 polymorphism of either G (IM12) or D (IM10, IM13) has evolved repeatedly in outgroup flies [25], indicating it is likely key for IM10-like peptide activity. (TIF) [file ppat.1009846.s003.tif]

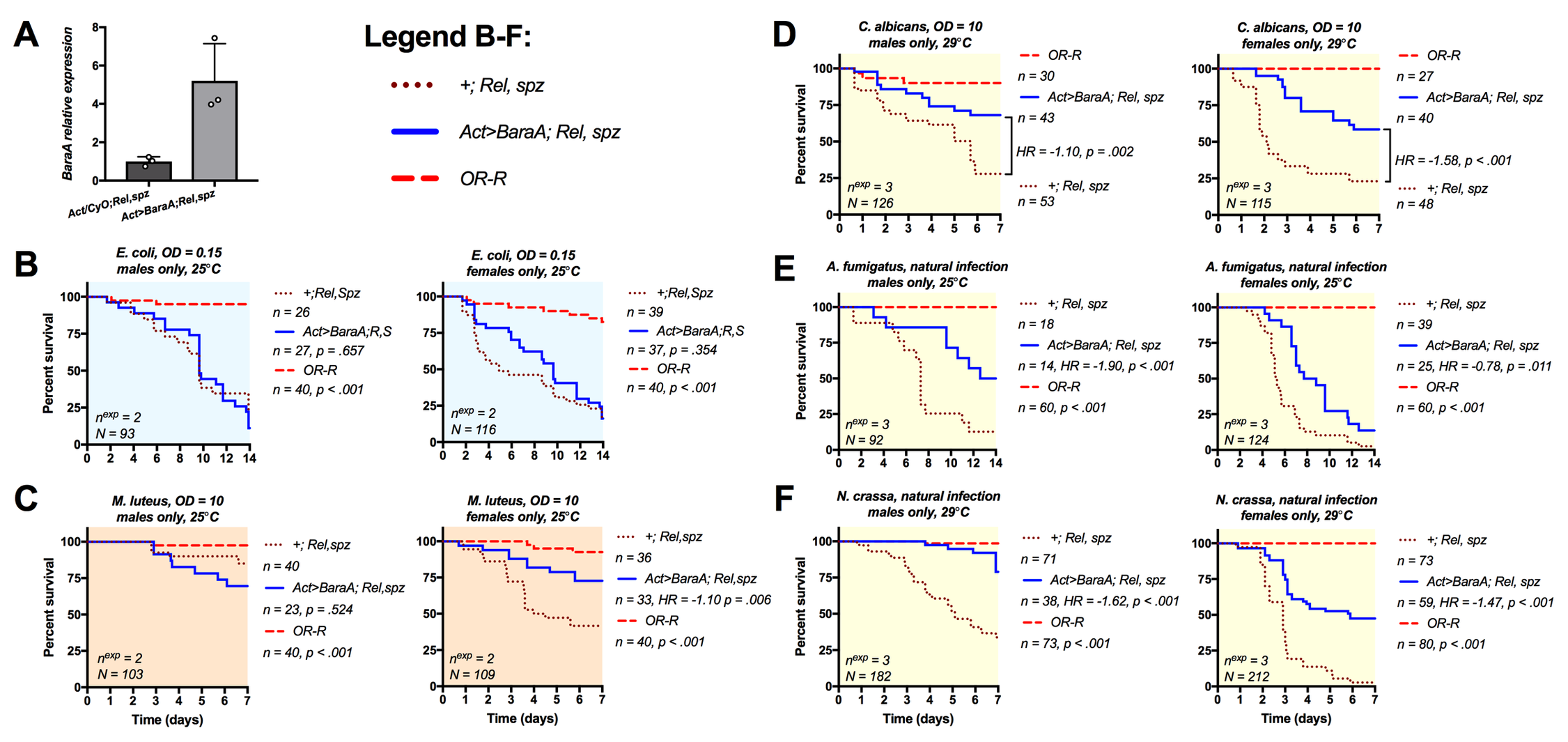

Supplement: S4 Fig — A) Validation of the UAS-BaraA construct in the Rel, spz background. Flies were unchallenged. B) Overexpressing BaraA did not improve the survival of Rel, spz flies upon E. coli infection. C) Overexpressing BaraA only marginally improves survival of Rel, spz females, but not males, upon M. luteus infections. Infections using a higher dose (OD = 100) tended to kill 100% of Rel, spz flies regardless of sex or expression of BaraA, suggesting that if BaraA overexpression does affect susceptibility to M. luteus, this effect is possible within only a narrow window of M. luteus concentration. D-F) Overexpressing BaraA improves survival of Rel, spz male and female flies upon injury with C. albicans (D) or natural infection with A. fumigatus (E) and N. crassa (F). P-values are shown for each biological sex in an independent CoxPH model not including the other sex relative to Rel, spz as a reference. (TIF) [file ppat.1009846.s004.tif]

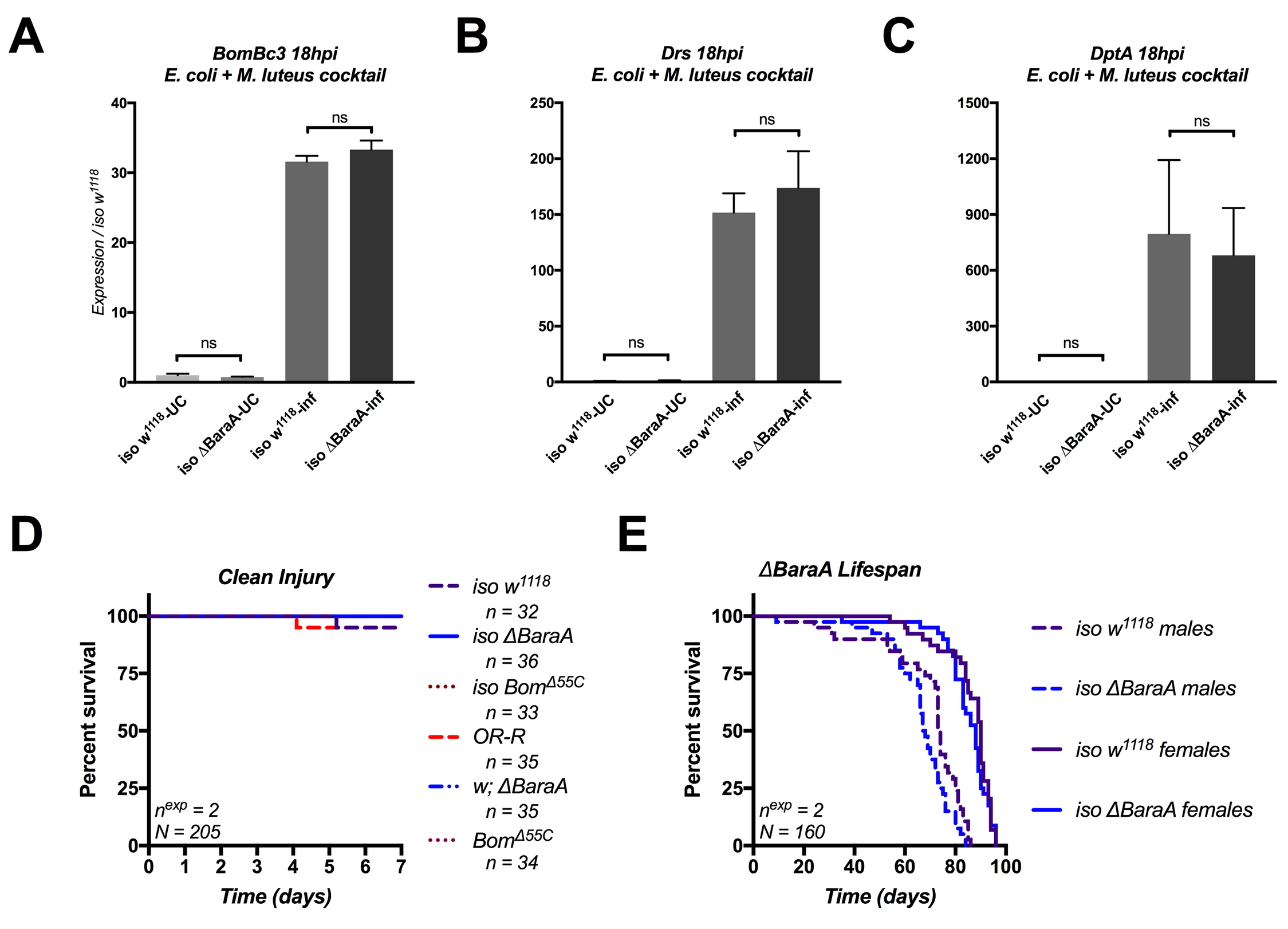

Supplement: S5 Fig — RT-qPCR shows that the expression of BomBc3 (A) Drs (B) and DptA (C) is wild-type 18hpi in iso ΔBaraA flies. D)BaraA mutants survive clean injury like wild-type flies. E) iso ΔBaraA flies have similar lifespan compared with the iso w1118 wild-type (males + females, iso vs. iso ΔBaraA: HR = 0.26, p = .118) (TIF) [file ppat.1009846.s005.tif]

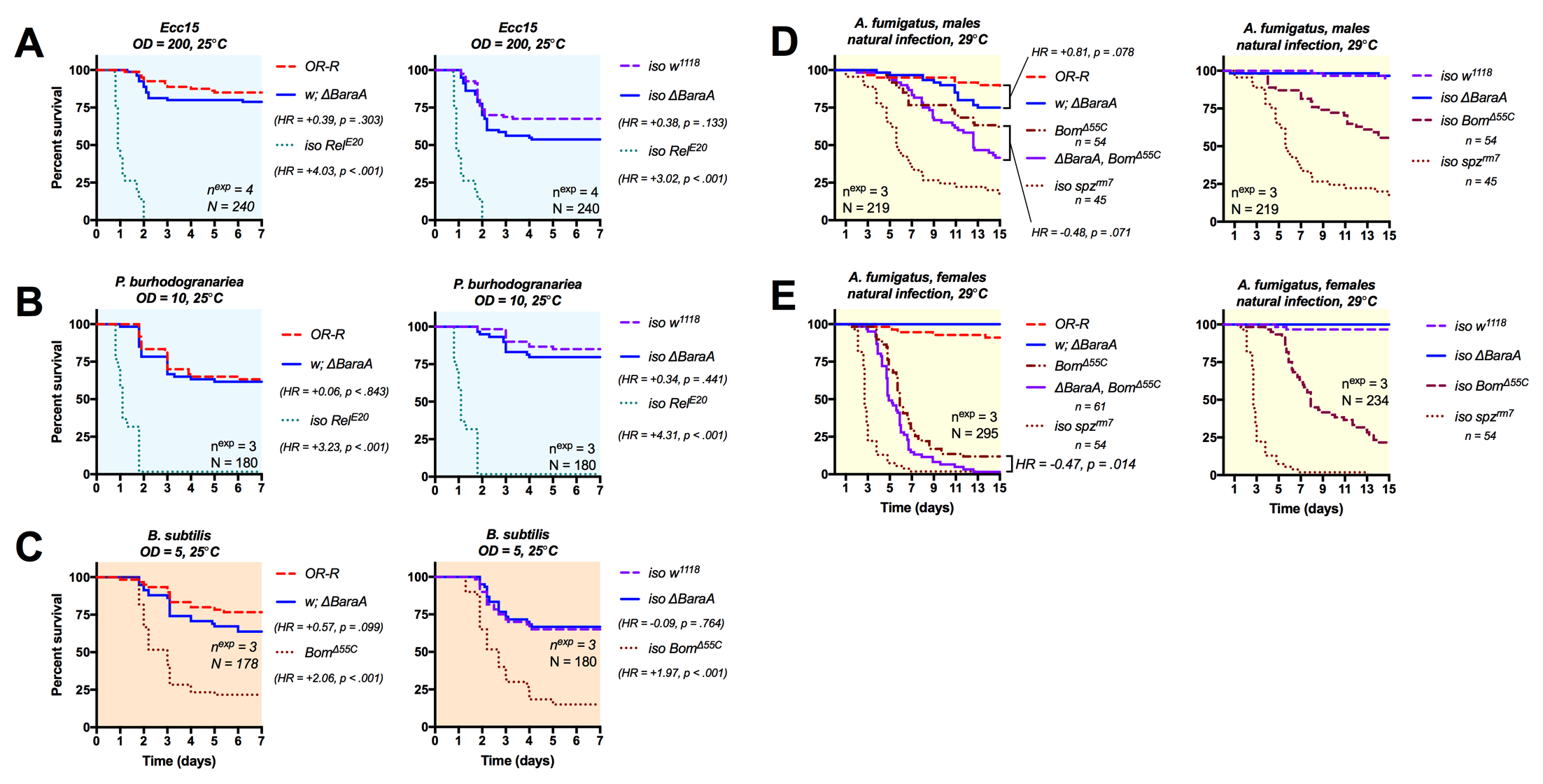

Supplement: S6 Fig — A-B) No significant susceptibility of ΔBaraA flies to Ecc15 (A), P. burhodogranariea (B), or B. subtilis (C), bacterial infections. D-E) w; ΔBaraA males were slightly susceptible to A. fumigatus natural infection (HR > 0.5, p = .078), but not females, nor isogenic flies. Additional infections using ΔBaraA, BomΔ55C double mutant flies reveals that BaraA mutation increases the susceptibility of BomΔ55C flies in both males and females (cumulative curves shown in Fig 5A). Blue backgrounds = Gram-negative bacteria, orange backgrounds = Gram-positive bacteria, yellow backgrounds = fungi. (TIF) [file ppat.1009846.s006.tif]

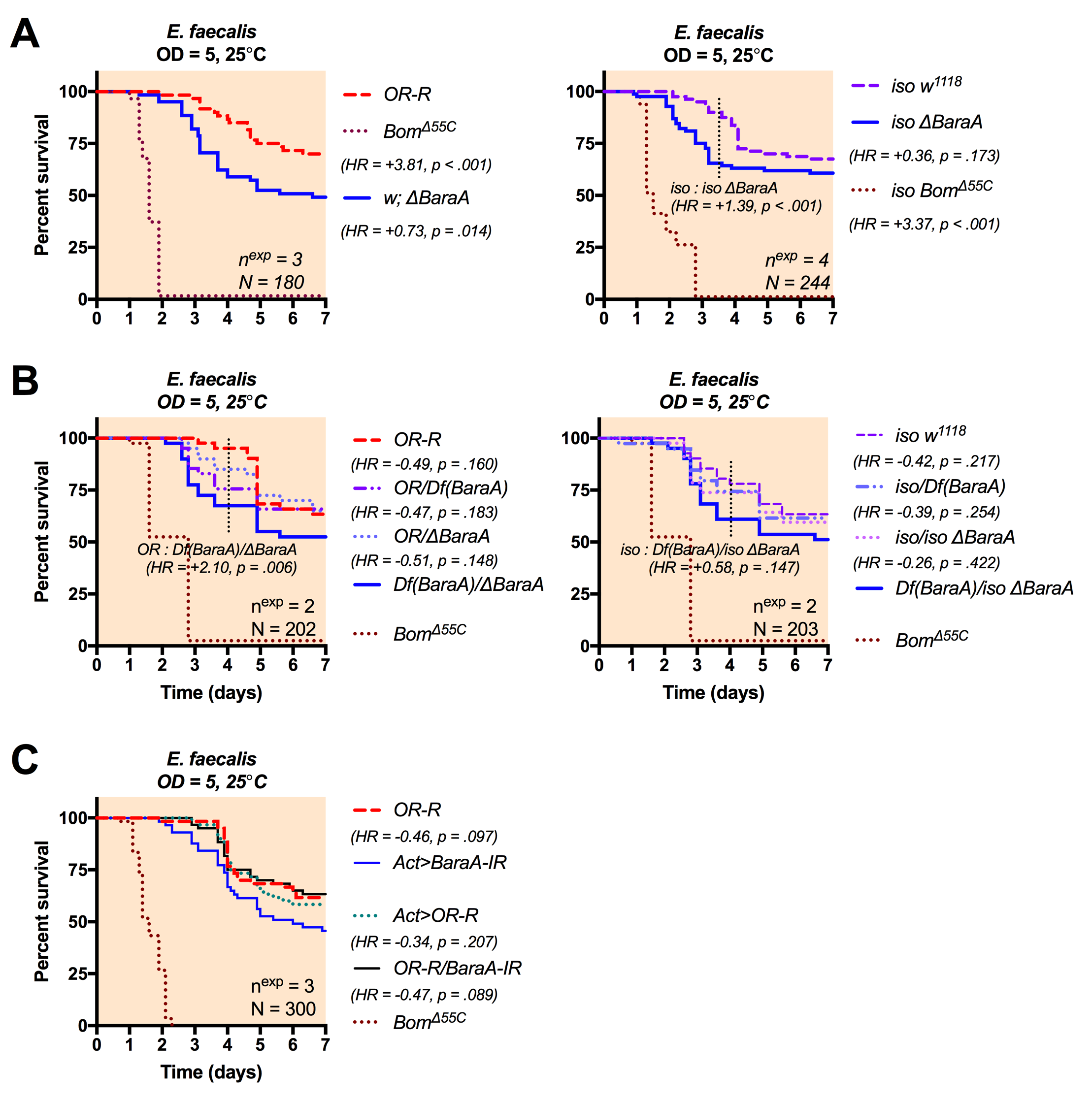

Supplement: S7 Fig — A)w; ΔBaraA but not iso ΔBaraA flies are significantly susceptible to E. faecalis. However we note that iso ΔBaraA flies suffer an earlier mortality than iso w1118 wild-type controls that is highly significant if the experiment is artificially censored at 3.5 days (dotted line and associated statistics). B) Crosses with a genomic deficiency (Df(BaraA)) leads to increased susceptibility in both the w background and isogenic DrosDel background, with Df(BaraA)/ΔBaraA flies suffering the greatest mortality in either crossing scheme. Both deficiency crosses yielded an earlier susceptibility in BaraA-deficient flies (shown with dotted black lines), however neither experiment ultimately reached statistical significance. C) BaraA RNAi flies (Act>BaraA-IR) suffered greater mortality than Act>OR-R or OR-R/BaraA-IR controls, but this was not statistically significant at α = .05; p-values reported are comparisons to Act>BaraA-IR flies. (TIF) [file ppat.1009846.s007.tif]

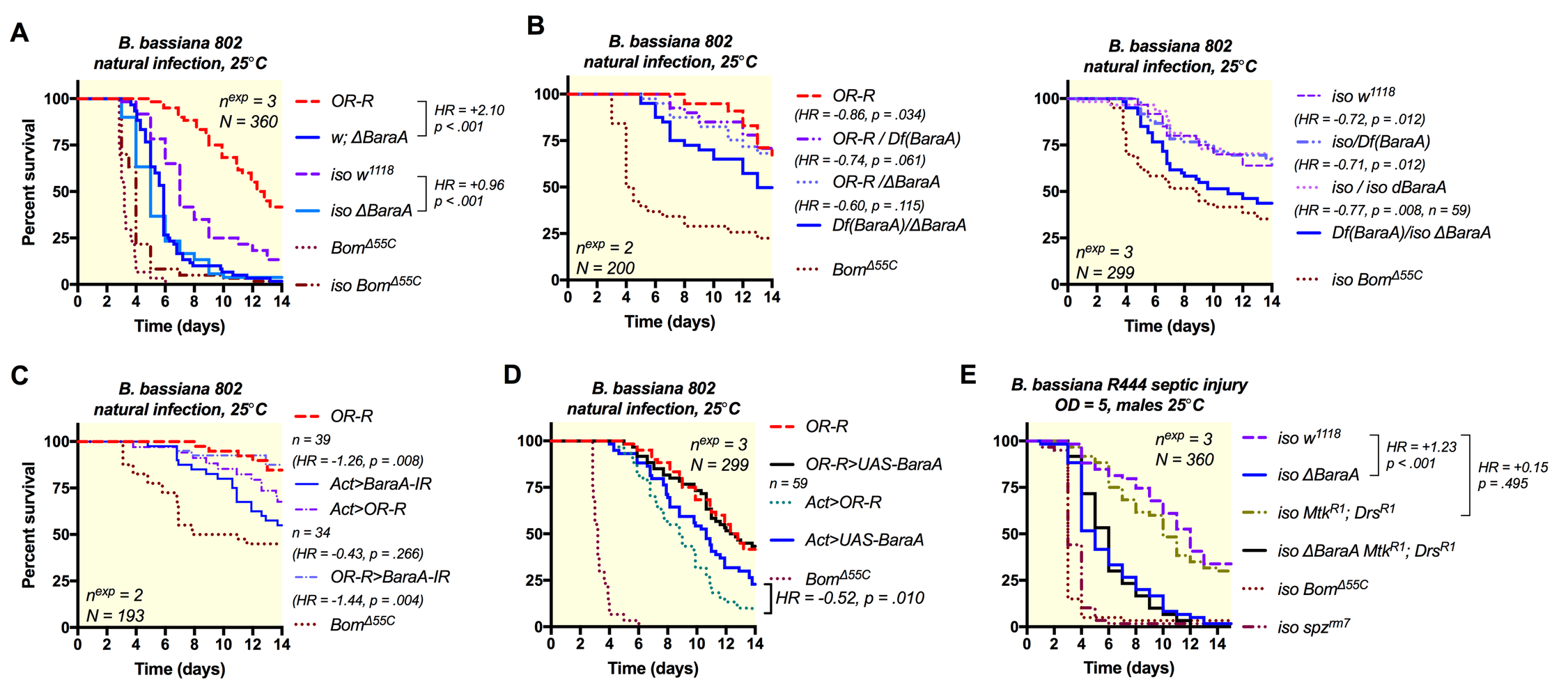

Supplement: S8 Fig — A)BaraA mutants in both backgrounds are highly susceptible to natural infection with the entomopathogenic fungus B. bassiana 802. B) Crossing with a genomic deficiency (Df(BaraA)) leads to increased susceptibility of Df(BaraA)/ΔBaraA flies for both the w background and isogenic DrosDel background relative to wild-type controls (p < .05) upon B. bassiana 802 natural infection. C) Act>BaraA-IR flies were more susceptible than the OR-R wild-type (p = .008) and OR>BaraA-IR (p = .004), although not significantly different from our Act>OR-R control (p = .266). D) Overexpressing BaraA (Act>UAS-BaraA) improved survival against B. bassiana 802 relative to Act>OR-R controls (HR = -0.52, p = 0.010). E) BaraA alone contributes to survival against B. bassiana to a far greater extent than the two canonical antifungal peptide genes Mtk and Drs, which in fact had little effect on survival outcome. (TIF) [file ppat.1009846.s008.tif]

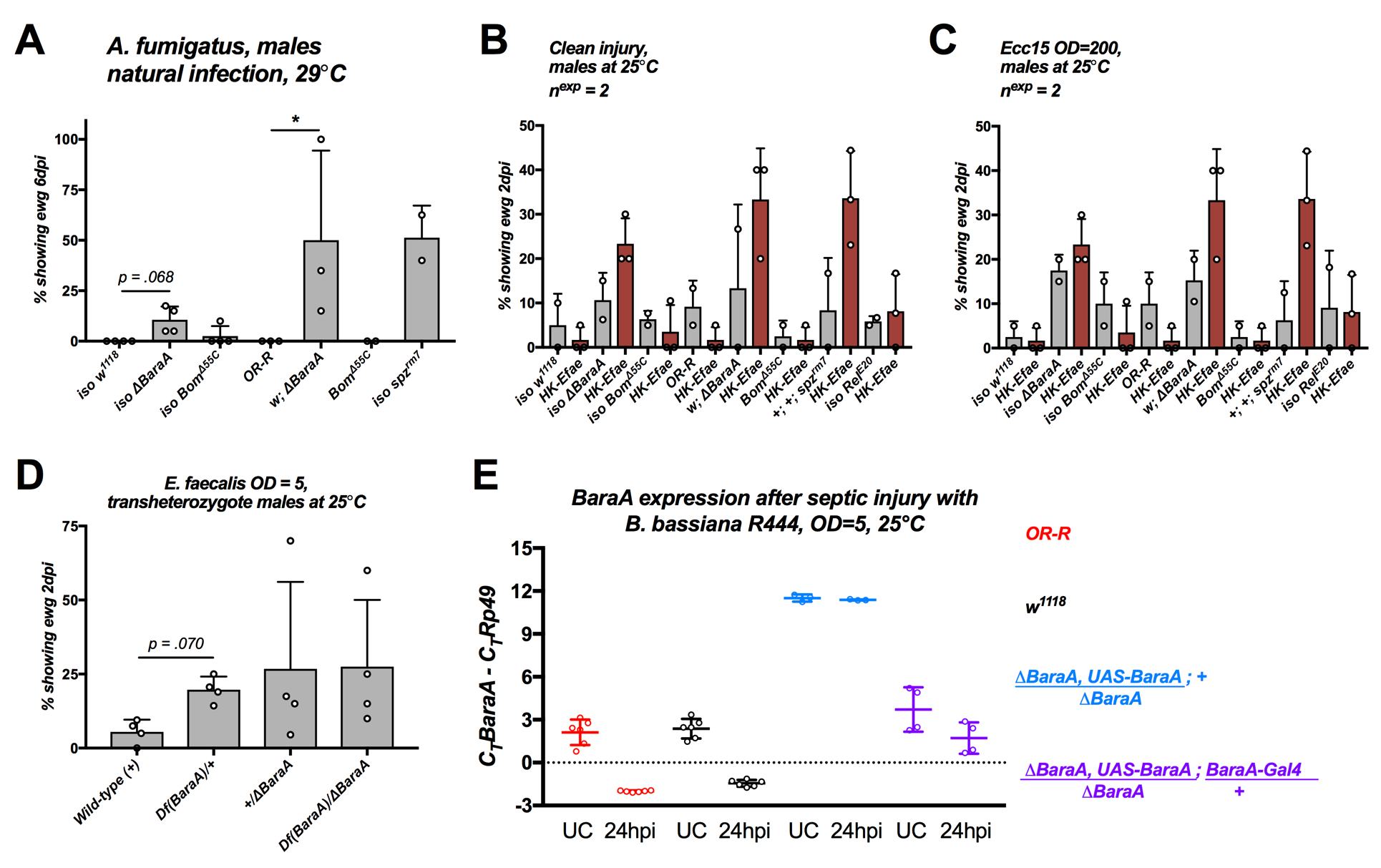

Supplement: S9 Fig — A) Erect wing occurs in flies given natural infection with A. fumigatus, wherein flies do not readily succumb to infection (S6D Fig) and no thoracic injury was introduced. B-C) Erect wing frequencies 2dpi after clean injury (B), or Ecc15 septic injury (C). The erect wing frequencies of flies pricked by HK-E. faecalis (Fig 6C) are included in brown to facilitate direct comparison with the frequency observed upon Toll pathway activation. D) The frequency of erect wing display is increased following E. faecalis septic injury in ΔBaraA/+ or Df(BaraA)/+ flies. Data points are pooled from w; ΔBaraA and iso ΔBaraA crosses after E. faecalis infections shown in S7A Fig and data in S1 Table. E) CTBaraA-CTRp49 (ΔCT) non-normalized expression of the BaraA-Gal4>UAS-BaraA method to better visualize expression level differences. This Gal4/UAS approach rescues BaraA expression in ΔBaraA flies, though not quite to wild-type levels. A very low level of expression was observed in ΔBaraA, UAS-BaraA/ΔBaraA flies without the Gal4 (indicating a tiny level of UAS leakiness), while BaraA was never detected in w; ΔBaraA flies. Differences in this ΔCT y-axis effectively equate to Log2 expression differences. The level of BaraA induction in these ΔBaraA, BaraA-Gal4>UAS-BaraA was ~3.3x the unchallenged state by 24hpi. (TIF) [file ppat.1009846.s009.tif]

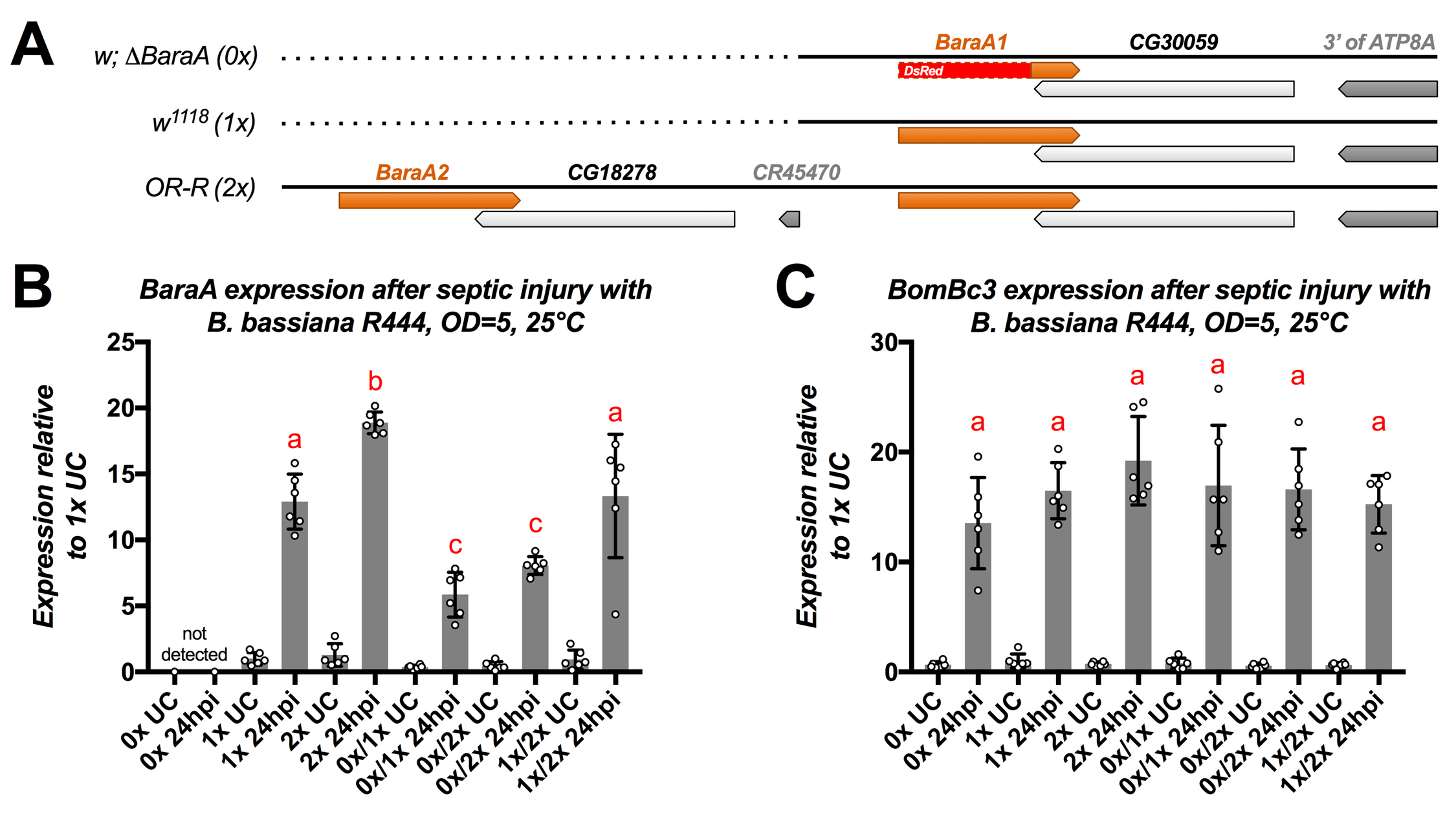

Supplement: S10 Fig — A) Schematic detailing the BaraA loci of genotypes used in transheterozygote crosses. B-C) BaraA (B) and BomBc3 (C) expression after B. bassiana pricking in BaraA homozygous or heterozygous flies. Transheterozygotes with one mutant locus have significantly reduced BaraA expression. Intriguingly, OR-R flies (homozygous for 2 gene copies) have higher BaraA expression levels compared to w1118 (1 gene copy) after infection (B), which appears to be unrelated to the activation of the Toll response generally as BomBc3 levels were comparable across genotypes (C). Instead, OR-R flies seemingly reach a slightly greater absolute expression (S9E Fig). Statistically significant differences at 24hpi are indicated by red letters, to facilitate complex multiple comparisons (one-way ANOVA with Holm-Sidak’s multiple test correction). Genotypes with the same letter group are not significantly different from each other. In all cases, no significant differences were observed amongst unchallenged flies. (TIF) [file ppat.1009846.s010.tif]
