# Supplementary material for: The Drosophila Baramicin polypeptide gene protects against fungal infection
Source: PLoS Pathog. 2021 Aug 25;17(8):e1009846. doi: 10.1371/journal.ppat.1009846 (PMC8423362; doi:10.1371/journal.ppat.1009846)
Supplement: S1 Text — (DOCX) [file ppat.1009846.s012.docx]

**Supplementary information:**

***Identification of the BaraA C-terminus as IM22 from Uttenweiler-Joseph et al.***

In 1998, Uttenweiler-Joseph et al. [8] described 24 immune-induced molecules by MALDI-TOF and informed predictions suggested that *BaraA* could encodes several of them [24]. We generated a knock out mutant for the *BaraA* gene (*BaraA^SW1^*), which we validated by MALDI-TOF peptidomic analysis. Strikingly, we noticed an immune-induced peak at ~5981 Da in Linear mode collections that is absent in *ΔBaraA* flies (**Fig** 2A); this mass closely resembled the 5984 Da estimated mass of IM22 from Uttenweiler-Joseph et al. [8], for which sequence was never determined. We took the Linear masses reported for then-unknown IMs from Uttenweiler-Joseph et al. [8] and post-hoc generated a standard curve with now-confirmed mass values from Levy et al. [24]. Our post-hoc standard curve corrects the mass of IM22 as found in Uttenweiler-Joseph et al. [8] to be 5973.5 Da. Using the same approach with our own linear data we find a mass of 5975.1 Da for our 5981 Da peak (S3 Data). With LCMS proteomics, we confirmed that the *BaraA* C-terminus is cleaved to remove 4 N-terminal residues, which should produce a putative 5974.5 Da peptide (**S2 Fig**). Together these observations indicate the *BaraA* C-terminus encodes the following 53-residue mature peptide, matching the estimated mass of IM22: ARVQGENFVA RDDQAGIWDN NVSVWKRPDG RTVTIDRNGH TIVSGRGRPA QHY.

The *BaraA* gene is therefore involved in the production of over one third of the classical *Drosophila* IMs from Uttenweiler-Joseph et al. [8], including: IM5, 6, 8, 10, 12, 13, 20 (doubly-charged IM24 [24]), 22, and 24.

***Sequence of the BaraA-Gal4 promoter construct***

The following 1675bp sequence was cloned from the DrosDel isogenic background into the pBPGUw vector to drive a downstream Gal4 gene, and inserted into the VK33 attP docking site using BDSC line #24871:

*Dif/dorsal binding site* **(bold)***:* **GGGHHNNDVH**

*Rel binding site* (underline): GGRDNNHHBS

>iso_DrosDel_BaraA_promoter-Gal4

CTGCTACTCCTCTACACATTCGACTCCTTCGCCTTGCTGGCTGGGAAAAAATTTTGCATAATTTATGTGGGTGCCGCGCACACGGAGGTCCCGACGGATTCGAAGTATCCGAAGGATTCGAAAGGAAAACAACGCACGAGCACCACGGCCAACTGATTTAAATGCAATTGCACTGAAGTATTTTGTTTGGCGAACGAAGCTGGATGAAATAGGGGGGTGTGGGGTTTTCTATTGAGACATCTGCACGTGCAACCGGAAACATCCGAAGAGAACAGCACAGGCCGGGCTACGCCGGGCAATTTCTTTTCATTTGCCAAGGTGTTGAGTTGCACCAACATTCGACATCGACGTGGCCAGAAGCCAACAAAAGCCAAGAGCCAAACCCCTTTTTGTGGTCACAAGTGTCGTCTATTTGTCGTGGGCATCTTGGGCACCTTGGGCATCCTCGACATCCTTGCCATTTTGGTCTGGCCAAGACAAACAACCAGCAAATTTAGTGTATTTTGTGCATTTTTAAAATTGTCCAAATTTATGTGACACGCTGCGCCAATTGATCAGATTAAATAAACATGAGGCCAAGCGAATCGAATTTGGCTTCACCAAGAAGACAATGCAGTCTGTATTCAAATGGGTGGGCGCATCCACCAAGCGGTGAATACAGTGACCGCTCGCTATAATGGACGGTCAGGTGTTACTTTAACTTAAAAAAATATGTAACAAATCTTATCAAGTTTGAAATAGATTGAAATAGATTTGGTTATTGCATTCGAAAGATATATATTAAATTCGAATATTCCAAGAAATTTCATGAGAATGTCACTTATGTCATGAGATTATATTAACGTACGAATAAACAATGTATTTTCCAAAATTAAAAATAAAATTTAATTTAATTACGCAGTACCTTTACACTATCAGTCGGAGGTAATAACTCATATAATTAGATTAGCATTAGATTTTAAAGCGAAAAACACTTAAAAGCTGAAATTATTAGACAACACTCTTAAATTAGTCGAGCTGATATATAGCCTCAAGTTTTGCTTAAATCCAAAGATAAAGGAATGCCTTCAAAAATATATTTTGTTTTATACCAAGTGACAGCAGAGAATGGGGTTGCAATATCTTAAAAGAGTTTCACTTAGCCAATATTTACTGCCATTGTTGGCCACCAAATAGTAGCAACCAGAGACTTCCAGGAATATATTCTCGTGTCAAATGCAATCCACTTTAAATGCAACTATCTGGCGGCTAAGAAAACCCGACAGTTTGATTCAAGTCGACGAAACAATATAAGCACGTGCTAAATAAAGAGACCTATGCAGTTAATACTCTTGTCATATTATAATATAATTTAGTGACATAAGTTGCATGGTATACGAGTACTGAACAAGTTATGGCAGCTTTTCCAAATAAGCGATCACATATTCCGCGGGATGATGGGTGGATTTCTAGCATATGTGGATGCTTAATGGCTTATTGC**GGGTCAGGGC**GGCGCAATCTGTTC**AGAAATTCCC**GAACGCACACCCATTTCAGATCAGATTGTGACGTTTT**GGGAAATTCT**TGACGATCGGTGTAAACAAGCTCAGCAACCAGATTCGATGGCTATTTGCCGGCTATAAATACTAGAAACCATTCGATTGCACTCAGTTGAAGCTGGGCTCTGGAACAGATCACA
