# Supplementary material for: The Drosophila Baramicin polypeptide gene protects against fungal infection
Source: PLoS Pathog. 2021 Aug 25;17(8):e1009846. doi: 10.1371/journal.ppat.1009846 (PMC8423362; doi:10.1371/journal.ppat.1009846)
Supplement: Attachment — Submitted filename: BaraRevCom_Point by Point Response-Jun18-2021.docx [file ppat.1009846.s018.docx]

| **Date:** | 2021-03-01 04:37:37 |
| --- | --- |
| **Last Sent:** | 2021-03-01 04:37:37 |
| **Triggered By:** | Redacted |
|  |  |
| **BCC:** | Redacted |
| **Subject:** | Review Commons Reviews for Refereed Preprint RC-2021-00650 |
| **Message:** | March 1, 2021  RE: Review Commons Refereed Preprint #RC-2021-00650  Prof. Bruno Lemaitre EPFL Global Health Institute Station 19 SV 3838 Lausanne, Vaud 1015 Switzerland   Dear Dr. Lemaitre,  Thank you for sending your manuscript entitled "The Drosophila Baramicin polypeptide gene protects against fungal infection" to Review Commons. We have now completed the peer review of the manuscript. Please find the full set of reports below.  Note: since the workflow of Review Commons is new, we are more than happy to schedule a call with you in case you have further questions about the content of the reviews or about the next steps, including the transfer of the reviewed manuscript to one of the affiliate journals. Please just let us know by return email and we will schedule an appointment.  To access your manuscript online and proceed to the next steps, please use the link below:  Link Not Available   NEXT STEPS:  1. RESPONSE TO REVIEWERS: You have *four weeks* to upload to the system an *initial* response/revision plan to your reviewed manuscript (please use the link above). If you need more time to draft this document, please let us know. At this stage, a full revision is NOT expected! You have nevertheless the option to upload a preliminary revised version of the manuscript (e.g. to fix minor points or textual changes). If you prefer to do so before forwarding your manuscript to one of our affiliated journals; please ensure that all changes are visibly tracked in the file.  2. POSTING THE REVIEWS ON BIORXIV or MEDRXIV: We strongly encourage the posting of your reviews on BioRxiv and MedRxiv. You will be able to instruct Review Commons to post the reviews and your response alongside your preprint. Please follow the instructions that are provided online on the submission screen.  3. TRANSFER TO AFFILIATE JOURNALS: When you submit the response/initial revision plan (see also https://www.reviewcommons.org/authors/#initial-revision), you will be presented with a screen that allows you selecting the affiliate journal to which you want to submit. Please note that the full set of reviews will be transferred alongside your response and the original manuscript. If you elected to upload an initial revised manuscript, it will be transferred alongside the original version.  4. EVALUATION AT AFFILIATES: Upon transfer, the recipient journal will consider the manuscript as a formal submission and use the transferred reviews and your response/revision plan to make an initial decision. Note that while it is under consideration at an affiliate journal, the manuscript cannot be transferred to any other affiliates.  5. NEW ROUND OF TRANSFER: If the first journal declines, you will be directly informed by this journal. The manuscript will then be unlocked on the Review Commons site so that you can initiate up to two additional rounds of transfer.  Please consult our detailed Guidelines for further details on the process and policies of Review Commons at https://reviewcommons.org/authors . If you have any further questions, please contact me directly.  Thank you again for sending us your work. We hope you will find the reviews useful and the Review Commons helps to disseminate your peer-reviewed research faster and more efficiently.  Best wishes,  Sara Monaco -- Sara Monaco, PhD Managing Editor Review Commons    ------------------------------------------------------------------------------ Reviewer #1 (Evidence, reproducibility and clarity (Required)):  **Summary:**  The authors use the fruitfly Drosophila melanogaster as a model to study innate immunity. In this manuscript, they study the effects of a set of antimicrobial peptides (AMPs) that are produced by furin cleavage of a larger precursor (Baramicin A, BaraA). Bara A is immune-induced in a Toll-dependent manner and has antifungal activity. Somewhat in line with expression in non-immune tissues, BaraA mutants show ab erect-wing phenotype in males.  **Major comments:**  The experiments are well-presented in a reproducible and statistically sound way. In particular care is taken to control effects of the genetic background. The immune phenotype of BaraA mutants is somewhat subtle but convincing and in line with recent findings by the same authors that some of the recently created CRISPR/Cas mutants in antimicrobial peptides have broader effects while others target intruders in a more specific manner or in combination with other AMPs. These are very relevant studies, which provide a balanced view of innate immunity in particular AMP action. I have one comment about the (BarA dependent, male-specific) erect wing phenotype: this is an interesting observation, which could stimulate work by others, I guess this is one reason why it was included in the manuscript. On its own it stands out a bit in the manuscript since in contrast to other parts, where insight into the underlying mechanisms is provided, this is not the case for the erect wing phenotype. The authors speculate about the non-immune expression, which may be responsible. One might use tissue-specific knockdown or rescue to check up on this (wing muscle or nervous system). This would be cost effective but delay publication for a few months. It depends a bit on the respective journal policy and the plans for further investment of the groups involved whether the phenotype is considered part of BaraA pleiotropism (which I could buy) or is considered too descriptive and should be used later for a later publication. Along similar lines, while sex-specific immune phenotypes are highly interesting, they open up many discussions about the underlying causes, both proximal and ultimate.  We agree the erect wing phenotype sticks out a bit in the manuscript. We included it because it is a consistent and surprising phenotype that we believe will spur future work and attention to this outcome to infection, which is driven by BaraA presence but likely also other factors.  We have now performed a set of rescue experiments to test the ability of systemic expression (driven by a fat body-Gal4) or BaraA-Gal4 driven expression of UAS-BaraA in a BaraA mutant background. We were both surprised and pleased to find that systemic expression in the fat body appears to rescue the presentation of erect wing in BaraA mutants. This runs counter to our earlier speculation that this was a neural-derived expression phenotype, but provides valuable insight into the nature of erect wing.  **Minor comments:**  The experiments look sound and previous work is mentioned sufficiently. The experimental design and results are easy to follow. I have mentioned some concerns about the erect wing phenotype (see above). Is there any evidence for metabolic regulation of BaraA (TF binding sites for example) in particular in the promoter fragment used for the reporter line? Did any of the fat body drivers show the same effect as the ubiquitous actin driver (this would increase specificity).  We now include a fat body driven rescue of the erect wing phenotype, and BaraA-Gal4 driven rescue of mutant susceptibility to B. bassiana infection.  For the reviewer’s interest: using a degenerate FoxO binding motif (Becker et al., 2010), we find two putative motifs within 1kb of the TSS using the FoxO motif TTGTTTAY. One within 130bp, and the other ~800bp from the TSS. It is an interesting question whether BaraA is regulated by metabolic signalling (or perhaps even vice versa). However we feel introducing the concept of metabolic regulation here is beyond the scope of the present manuscript.  Why was pimaricin used, it seems presently as a representative of membrane-active antifungal drugs, which BaraA peptides are likely not. Still, using combinations with other insect (Drosophila) antifungal AMPs would be more physiological, maybe this was tried and did not work, but should still be discussed. Or do the authors want to imply that physiologically the Daisho peptides or Bomanins have this effect? Perhaps elaborate on this.  Pimaricin was used as a commercially available antifungal with a well-characterized membrane-disrupting activity. Pimaricin has a reversible binding activity to ergosterols that inhibits transport of amino acid and glucose across the membrane, leading to temporary loss of membrane integrity and increased passive transport. This mechanism is general to all fungi, making it a useful commercial product to test for synergy using both yeast and filamentous fungi like Candida and Beauveria. Drosophila-derived antifungals are not as generalist (e.g. Mtk in PMID: 28811531), or have complex 3D structure and aren’t readily available for purchase (e.g. Drs). We did also test Hyalophora Cecropin (Sigma) against C. albicans (no effect in vitro in our hands). We have restructured the language in lines 259-on to better explain the logic of this assay. We also now discuss this point in lines 539 to suggest that use of insect antifungals may better synergize with IM10-like peptides.  In Fig. 1: part H is missing although mentioned in the legend.  Thanks for catching. Corrected.  In the abstract:  it should be more clearly mentioned that the erect wing phenotype was observed in the mutants. line 27 and 28, replace one "characterized" line 28: contribute line 33: entomopathogenic  line 68: AMPs  Each point is now addressed  Reviewer #1 (Significance (Required)):  **Significance:**  The evolutionary relevance and therapeutic potential of AMP synergism is an emerging topic both within insect immunity, innate immunity in general and its use in patient treatment [1, 2]. The latter aspect may be interesting to justify the use of pimaricin. Thus, the work presented here in combination with previous work from the authors leads to a more balanced view of the action of insect AMPs (the authors call that the logic of the Drosophila effector response) with implications for human innate immunity and perhaps even therapy of diseases. Therefore, the data will be interesting for a broad audience. The use of models such as Drosophila, which can be manipulated in a targeted manner has provided insights that are beyond the study of single AMPs in vitro. Still, using overexpression as done in some cases here should be interpreted cautiously and should - if available - compared to data on in vivo concentration of AMPs (the authors try to derive estimates from the MS data), which may be difficult in case there are large local differences.  **My own field:**  primarily insect immunity with a background in mammalian immunity, although I am not able to keep up with all recent development in mammalian immunity.   Reviewer #2 (Evidence, reproducibility and clarity (Required)):  **Summary:**  Hanson et al. investigated an antifungal gene they named BaraA, which codes for a protein that is proteolytically processed into 8 smaller peptides. BaraA expression is induced by Toll pathway signaling with minor input from the Imd pathway. It is expressed in the fat body upon immune challenge and expressed in other tissues such as head and eyes. Overexpression of BaraA increased the survival of animals defective in both IMD and Toll pathways. In vitro, the combination of the three major BaraA peptides displayed modest inhibitory effect on fungal pathogens when combined with the antifungal drug Pimaricin, however BaraA peptides alone showed little or no antifungal activity. BaraA deficient mutants showed little to no significant difference in bacterial resistance but appeared to show susceptibility to fungal infection; this fungal susceptibility was independent of the Bomanins. Male BaraA mutants also displayed an erected-wings phenotype when subjected to infection.  There are 3 key findings: - BaraA overexpression conferred protection against fungal infection. - BaraA-derived peptides displayed antifungal activity in vitro in conjunction with Pimaricin, in vitro - Loss of BaraA decreased fungal resistance.  **Major Concerns:**  The results from the overexpression experiments were clear. However, the second and third findings were less convincing. - The cocktail of IM10-like BaraA peptides showed significant synergy with Pimaricin in killing C. albicans at only one dose out of the five tested, and this combination has modest (19-29%) inhibition on hyphae growth of B. bassiana. The in vitro antifungal experiments might be more compelling if other fungi were examined and/or combinations with other antifungals were investigated, where synergy might be more robust.  We agree the in vitro effect is modest. We have twice-repeated the C. albicans doses focused on16ug/mL, which shored up statistical significance (Pimaricin vs IM10-likes + Pimaricin: formerly p = .108, now p = .016 in one-way ANOVA). We also now suggest that future studies should establish different in vitro approaches that might better mimic physiological conditions. We have also softened our wording regarding the hypothesis of IM10-likes potentially acting intracellularly, as this mechanism should be tested rigorously and alternate mechanisms ruled out in a future biochemical study (e.g. Line 562 we now explicitly suggest future study to confirm the IM10-like mechanism of action).  Of note: we can now also report an additional trend reinforcing IM10-like peptides as immune agents. In a separate study (Hanson and Lemaitre, 2021; see bioRxiv preprint below), we find various Baramicin genes with either immune or non-immune expression patterns. Strikingly, IM10-like peptides are consistently lost in non-immune Baramicin genes, but retained in all immune Baramicin lineages (see *Hanson and Lemaitre, 2021 Fig. 5C*). Conversely, the IM24 domain is preserved in non-immune orthologues, implicating the broad function of IM24 as being amenable to non-immune contexts. These observations reinforce IM10-like peptides as being useful specifically in an immune context, but dispensable in non-immune contexts, as would be expected of peptides with a primarily antifungal purpose.  *Hanson and Lemaitre. 2021. Extensive duplication and convergent sequence evolution of an antimicrobial peptide gene. bioRxiv, doi:* [*https://doi.org/10.1101/2021.02.24.432738*](https://doi.org/10.1101/2021.02.24.432738)  Finally, we also hope the ΔBaraA, BaraA-Gal4>UAS-BaraA rescue adds confidence to the specific importance of the BaraA gene products (which are primarily the IM10-like peptides) in defence against B. bassiana. This result is now shown in Fig. 4D.  - The most problematic issue with this data is the control of genetic background in the study of the BaraA mutant strains. Much of the survival data compares mutant strains (BaraA and/or Bom∆) with Oregon-R as a wildtype. As best we can tell, the BaraA and Bom strains are not in the genetic background and neither is particularly similar to OR-R. If the authors can justify the use of OR-R as the wildtype control for these experiments, they should do so explicitly. Otherwise, these experiments are very difficult to interpret. This issue is highlighted by other data, where genetic background is carefully controlled, in the iso-w background, and the survival phenotypes are much more mild, and do reach significance is some infections, by log-rank analysis. All experiments should be performed in this controlled background to enable firm conclusions and interpretations.  We agree the use of OR-R is arbitrary. We have modified the text to state this explicitly (e.g. Line 301-310, and 632-642). We also explain the issue more clearly (we hope) in lines 301-310. The key point is that we were forced to arbitrarily mix the *w; ΔBaraA* background to restore a wild-type Dso2 locus, leaving no appropriate wild-type control for this background. However we feel it is important to obtain results in more than one background, and not rely solely on isogenic flies to validate the effect of a mutation. In our hands, we have had instances with both “gain of susceptibility” or “loss of susceptibility” upon isogenization, emphasizing the importance of using different genetic backgrounds even if one is arbitrary.  Both to help with this issue and to save space, we have now combined the natural infection survivals into one panel, and moved this panel to Fig. S8A as the cross-experiment results are more consistent using our new *B. bassiana* septic injury data. We also show an additional wild-type (w^1118^) in Fig 4B-C, for which we have data, and have performed septic injuries with the isogenic background now which we hope shores up confidence in the relative importance of BaraA in defence against B. bassiana including in the iso background (Fig. 4B).  While the survivals with B. bassiana 802 are informative, we will note here that our intensities of infection varied over time as these natural infections relied on rolling flies on sporulating plates. i.e. Infection intensity varied by plate. This may provide useful context to interpret the apparent mortality of iso vs. OR-R wild types in now-Fig. S8A. After returning from the Covid lockdown, our B. bassiana 802 strain virulence was greatly reduced compared to before the lockdown. For this reason we were motivated to seek out a more standardized approach, ultimately leading us to use B. bassiana R444 commercial spores. We feel the B. bassiana R444 experiments are much more consistent across replicates (e.g. Fig. 4B, 4D), and we would lend greater weight to these experiments for genetic background comparisons than the B. bassiana 802 natural infections (though of course both agree in trend).  Finally, we also now include experiments using Metarhizium rileyi (a commercial fungal pathogen of noctuid moths). While this pathogen is slower-growing and not as virulent as B. bassiana, we nevertheless recovered a significant susceptibility of BaraA mutants (now-Fig. 4A). Consequently we have moved the E. faecalis results to the supplement, and now focus Figure 4 strictly on the antifungal role of BaraA.  **Minor comments:**  - Figure 1A mined data from a previous published study, which is acceptable, but this data presentation lacks proper description of the methodology, reproducibility, and statistics.  We have clarified that the induction coefficient reports a value based on Log10-fold differences, later normalized to wild-type flies.  - The authors need to clarify the condition of the flies in Figures 1D to G (as well as S1C and D). Infected? Baseline? It is not clear.  Baseline expression now in the figure legend.  - There is no visualization of the genomic location of the BaraA deletion, which should be added to figure 2C.  We thank the reviewer for the suggestion. We have added a diagram of the BaraA deletion to Figure 2A. Figure 2C specifies the duplication takes place in the 50A gene region (of Chromosome 2R).  - The authors should include the full genotype information for the Bloomington stocks, since the BL numbers may change over time.  We have added this to supplementary data file 2.  - In Figure 2C, the authors should include some information about which lines possess the single BaraA locus and which lines have the duplication event.  We now cite our separate preprint, which has this information as a main finding of that study.  - The author should elaborate on what is known about Dso2 and how the aberrant Dso2 locus might affect their assays. The info here is incomplete and confusing.  We hope we have clarified what transpired (above for reviewer 1). The aberrant Dso2 locus does not affect any of the data included in the study, as we have only used flies with a wild-type Dso2 locus. This information is included to indicate that there is no appropriate background control for w; BaraA flies, as these flies were recombined once to remove the aberrant Dso2 locus with an arbitrary background. We have modified lines 301-310 to better explain this issue.  - Does the Ecc15 strain used in the paper innately resist Ampicillin? If yes, then the result of Ecc15 resisting the combination of IM cocktail and Ampicillin does not reveal much.  The Ecc15 strain was susceptible to Cecropin. Ampicillin was used for E. faecalis. We have included these experimental data below but we did not include them in the manuscript to better focus the discussion.    ***Figure legend: no activity of IM10-like peptides against Ecc15 or E. faecalis alone or in combination with membrane-disrupting antibiotics.*** *Here we show the mean killing activity from two experimental replicates that we trust to be representative. The concentrations of antibiotics are indicated along the bottom, and the treatment legend at the top. Blue lines used Ecc15 and red lines used E. faecalis. Use of IM10-like peptides in combination with ampicillin seemed to inhibit the ability of ampicillin to kill E. faecalis. IM10-like peptides alone (horizontal dotted lines) also caused slightly higher E. faecalis colony counts (seen here as a negative killing activity). We have no explanation for this given that, if anything, BaraA mutants were slightly susceptible to E. faecalis (also found in a separate preprint (Huang et al., 2020; doi: https://doi.org/10.1101/2020.11.23.394809). As such, we suspect the minor susceptibility of BaraA mutants to E. faecalis arises from an independent mechanism from the susceptibility to B. bassiana, and suspect E. faecalis susceptibility may possibly be mediated by another peptide of the BaraA gene (such as IM24).*  - It is unclear what the concentration of pimaricin was used for Figure 3E.  This information has been added to Figure 3E legend.  - The authors should include a clear genetic explanation for their conclusion that BaraA and Bomanins function independently. The text describing this double mutant analysis could be more informative.  We have added text around line 411-420 to explicitly state our interpretation. We hope the revised interpretation is clearer.  - BaraA overexpression significantly improved female survival against M. luteus (Figure S4C, p=0.006), this is interesting but not mentioned in the text.  We clarify in the Fig S4 caption that this result is possible only within a narrow range of M. luteus concentration, and only in females. OD=100 example data are provided below. For context: OD=10 M. luteus is quite low, given that it is a larger coccus bacterium where CFUs/mL is ~30x less than E. coli given the same OD600 (in our hands).    ***Figure: no rescue of susceptibility to M. luteus using a higher concentration (OD=100).***  In general, we saw only minor Baramicin*bacteria interactions (most notably using E. faecalis infection). We thus prefer to be reserved in communicating our core message, and not suggest a prominent effect of BaraA on gram-positive bacteria. However we also do not want to wrongly rule out a contribution of BaraA to defence against Gram-positive bacteria given these disparate observations across bacterial species. We have moved E. faecalis data to the supplementary figures, replacing it with new Metarhizium rileyi data in line with including these data, but focusing our message on an antifungal role.  - The author should be clear and consistent about the pathogen source (lab grown vs. commercial) and method of infection (natural infection vs. septic injury). The authors should explain the difference in virulence between different infection models and methods.  We have included the pathogen, route of infection, OD (when septic injury used), temperature, etc… in the figures directly above each panel. We have now also included additional details for pathogens and strain info when available in supplementary data file 2.  We now emphasize virulence (e.g. Line 367, line 396, “mild” in Fig 5 caption) to emphasize the point that we use pathogens of appropriate virulence to reveal phenotypes given our infection models, which differ drastically in baseline susceptibility (Rel, spz vs Bom vs wild-type).  - The sex-specific erected wings phenotype is interesting, but does not contribute to the overall significance of the manuscript. The authors should consider moving Figure 6 to the supplement.  We agree that the erect wing phenotype is puzzling. We have now added support to this phenotype through a Gal4/UAS rescue, revealing that fat body-driven BaraA can rescue the phenotype. Recent studies have highlighted AMP interactions with the nervous system, and we hope in this light the BaraA erect wing phenotype is intriguing to a broad community. We have already had fly stocks requested to test the interaction of BaraA in the brain following a conference presentation. We hope this emphasizes that the phenotype is of significant interest to the community as a novel behavioural outcome of infection tied to a specific gene/pathway.  Reviewer #2 (Significance (Required)):  This work is a potential step in characterizing the immune effectors downstream of the Toll pathway that contribute to the Drosophila defense against fungal pathogens. These effectors so far have not been characterized and understood. We are familiar with the Toll pathway and its effectors, but in no way are experts.    Reviewer #3 (Evidence, reproducibility and clarity (Required)):  **Summary:**  Hanson et al. have set out to investigate the BaraA gene, and show that the gene encodes for several immune induced molecule (IM) peptides, namely IM10, IM12 (and its sub-peptide IM6), IM13 (and its sub-peptides IM5 and IM8), IM22, and IM24. Flies lacking BaraA are viable but susceptible to specific infections, notably by the entomopathogenic fungus Beauveria bassiana. Furthermore, they show that BaraA is antimicrobial and, when combined with the antifungal Pimaricin, it inhibits fungal growth. In principle, this is a nicely written paper with interesting findings. The authors show induction of BaraA with different micro-organisms and where BaraA is expressed, using a BaraA reporter. The exploration of the genomic area, showing the duplication of the BaraA locus is really nice work. Also, the survival experiments show quite clear phenotypes and therefore effects for BaraA.  **Major comments:**  Line 153, related results: Fold induction of BaraA is greater with E. coli (~50) than with C. albicans (~20) or M. luteus (~6) - any comments on this? Also, infection times with these microbes are different - some comments about BaraA kinetics? Based on Fig 1B, BaraA looks to be highly induced by E. coli, although in Fig 1C, after 60h, reporter induction by E. coli is much less than with M. luteus. Some clarification about the kinetics of BaraA in these different infection models is needed.  We initially used time points correlated with the peak of induction for each pathogen (Lemaitre et al., 1997), but agree and realize this doesn’t lend itself to a clear interpretation of the data. Here we will note that BaraA was still induced to about the same extent in Rel^E20^ flies (~50X relative to the Rel^E20^ UC baseline), but this looks like only ~25x when compared to the UC wild-type as the baseline. We suspect this induction is primarily due to injury, possibly regulated by the Toll pathway given that the induction still occurs in Rel mutants (also seen in the kinetics of Fig1A). We have now moved the E. coli qPCR to the supplemental figure and interpret the induction as a response to injury.  To address these concerns, we performed additional experiments using the BaraA>mGFP reporter and scored the kinetics of GFP intensity using unchallenged, E. coli, or M. luteus-pricked flies (new Fig. 1C). We also now cite a recent time course transcriptomic study that confirms BaraA’s expression profile upon pricking with E. coli LPS+PGN (Schlamp et al., 2021) (Line 146). This triggers a sustained Imd response, however Toll effectors like Drs, BomBc3, and BaraA resolve by 48hpi and are no longer significantly different from basal levels. Thus our study and theirs agree the early induction seen at 6hpi with Imd stimulus resolves quickly, and we quantify the cumulative mGFP signal, which is weak in our BaraA>mGFP flies when pricked with E. coli.  Erect wing phenotype in males: This is a bit surprising finding/interpretation. I have also seen erect wings in E. faecalis-infected flies, but I am not sure now in which flies I saw this; I have never tried to quantify this nor made any notes about females/males in this context. I normally use Myd88 RNAi (VDRC #25399) as a control in my experiments, and if they were the ones showing the erect wing phenotype in a prevalent manner, they would also lack BaraA (which is dependent on the Toll pathway function). At the time of doing my experiments, I just interpreted this in the way that the flies looked "sick", they were lifting their wings up and walking around rather than flying. When monitoring my survival experiments, I assumed that the ones with wings up were the ones dying next (the sickest). What is your interpretation; are the flies still ok or very sick, when this erect wing starts to appear?  We thank the reviewer for their interest in erect wing, and for sharing their anecdote. We do not currently suspect that erect wing predicts immediate death based on anecdotal observations of erect wing prevalence before and after periods of mortality. We have not done the necessary setup to track individual flies and confirm this. However we note that erect wing appears even upon heat-killed E. faecalis pricking, and so the phenotype is not directly related to pathogen dynamics but rather host dynamics.  We now include a supplemental figure (Fig S10) using BaraA mutant and wild-type transheterozygotes that shows that mutant/+ transheterozygotes have significantly reduced BaraA expression regardless of wild-type copy number. These results support the observation of increased erect wing in transheterozygotes in Fig. S9D as relying on BaraA expression, and not so much on pathogen dynamics.  **Minor comments:**  Wording: In the intro, line 78: "Many of the genes that encode these components of the immune peptidic secretome have remained largely unexplored." - I would say "had remained" until recently - especially the quite recent Bomanin work and work with Daisho1 & 2 have brought about a lot of new information about this "immune peptidic secretome".  Done. Now Line 79.  Fig 1A: What is BaraA called in DeGregorio et al? Can't find it (easily) from their lists. Please write BaraA into the Fig 1A graph, to make it clearer. Also, write somewhere in the text or Figure legend what the gene is called in DeGregorio et al (CG33470? CG18278? something else?)  BaraA2 (CG18279) was known as IM10 up until the Dmel_R6 genome release, when it was renamed as IMPPP. The name in DeGregorio et al. was IM10. Following our study and the one of our colleagues (Huang et al., 2020; bioRxiv), we have agreed on the name Baramicin to give it a name related to its function. We have added an emphasis on the CG numbers in Lines 105 and 125 upon introducing the BaraA genes to avoid confusion regarding this gene’s complex naming history.  The title above figure panel 1A has been updated.  Line 238 Reference to Supplementary data file 1: In the supplementary data files I downloaded, I can't see the files numbered as data files 1 and so on. Instead, there are folders (Fly stocks, NF-kappaB sites, Primers used) and the files have names. Please clarify that the supplement names match the text.  We now use a more systematic approach to organizing our supplementary data instead of packaging various folders in a .zip file. We thank the reviewer for bringing this to our attention.  Reviewer #3 (Significance (Required)):  - Describe the nature and significance of the advance (e.g. conceptual, technical, clinical) for the field. State what audience might be interested in and influenced by the reported findings.  I think the significance of this work is great for Drosophila immunity researchers. The nature and mode of action of many of the Toll pathway -induced peptides is not known, so more information on them is much appreciated by the field. Also, studying molecules with potential antimicrobial activities is also potentially interesting for wider audience.  - Place the work in the context of the existing literature (provide references, where appropriate).  The main Drosophila immunity pathways are the Toll and the Imd pathway, and when activated, several immune effector genes are induced. In 2015, a group of Toll pathway target genes was identified by mass spectrometry, that the authors here call "the immune peptidic secretome". (Clemmons AW et al., PLoS Pathogens 2015). Many of these peptide genes have been uncharacterized, although emerging studies have shed light to these findings in the past three years (Lindsay SA et al J. Inn. Imm. 2018; Cohen LB et al. Front.Imm. 2020). This research brings about new information on yet uncharacterized peptides in this group.  - Define your field of expertise with a few keywords to help the authors contextualize your point of view. Indicate if there are any parts of the paper that you do not have sufficient expertise to evaluate. Drosophila melanogaster, innate immunity, humoral immunity, cellular immunity Toll pathway, Imd pathway, immune-induced molecules |
