# Supplementary material for: The Drosophila Baramicin polypeptide gene protects against fungal infection
Source: PLoS Pathog. 2021 Aug 25;17(8):e1009846. doi: 10.1371/journal.ppat.1009846 (PMC8423362; doi:10.1371/journal.ppat.1009846)
Supplement: Attachment — Submitted filename: PPATH_Response_July26.docx [file ppat.1009846.s019.docx]

Dear editors,

Thank you very much for this positive news! Please find below our responses to reviewer comments (in blue).

Mark Hanson and Bruno Lemaitre.

***********************

Reviewer Comments (if any, and for reference):

Reviewer's Responses to Questions

**Part I - Summary**
Please use this section to discuss strengths/weaknesses of study, novelty/significance, general execution and scholarship.

Reviewer #1: I have already reviewed this manuscript once, as part of the Review Commons -system (as Reviewer #3). I feel that my concerns / questions / suggestions at that stage have been adequately addressed and I don't have any more suggestions.

Reviewer #2: The authors use the fruitfly Drosophila melanogaster as a model to study innate immunity. In this manuscript, they study the effects of a set of antimicrobial peptides (AMPs) that are produced by furin cleavage of a larger precursor (Baramicin A, BaraA). Bara A is immune-induced in a Toll-dependent manner and has antifungal activity. Somewhat in line with expression in non-immune tissues, BaraA mutants show ab erect-wing phenotype in males.

Reviewer #3: BaraA is a Toll-dependent gene expressed in the fat body upon immune challenge that codes for a protein that is proteolytically processed into 8 smaller peptides. Additionally, the BaraA locus is frequently duplicated in several lines of D. melanogaster and this gene is expressed in other tissues (head, eyes, etc.) at baseline. Male BaraA mutants displayed an erected-wings phenotype when subjected to infection, which can be rescued through overexpression of BaraA in the fat body.
There are 3 key findings:
- BaraA overexpression conferred protection against fungal infection.
- BaraA-derived peptides displayed antifungal activity in conjunction with Pimaricin in vitro.
- Loss of BaraA decreased fungal resistance, independent of the Bomanins.

**Part II – Major Issues: Key Experiments Required for Acceptance**
Please use this section to detail the key new experiments or modifications of existing experiments that should be absolutely required to validate study conclusions.

Generally, there should be no more than 3 such required experiments or major modifications for a "Major Revision" recommendation. If more than 3 experiments are necessary to validate the study conclusions, then you are encouraged to recommend "Reject".

Reviewer #1: (No Response)

Reviewer #2: (No Response)

Reviewer #3: The authors addressed and resolved many issues in previously draft. The additional results were clear and the use of various backgrounds was justified. However Lines 140-148 are very confusing. The authors claim BaraA is highly induced in the fat body by M. luteus but not E. coli, and triggered by the Toll pathway and not the Imd pathway, but S1B showed BaraA being induced strongly (45x) at 6hpi by E. coli, and lesser extent (6x) by M. luteus at 24hpi. And while the E. coli triggered expression is mostly independent of Relish, this experimental design does not rule out other components of the Imd pathway, nor did the data and the use of reference definitively prove that this is triggered by Toll in response to E. coli. The methodology of figure 1C, which indicates a more robust response to M. luteus (but not unresponsive to E. coli - the use of “not” in the text and figure legend is unjustified) is not explained in either the text or the legend. Further, the analysis in figure 1C appears to be only a binary call, based on a reporter over a timecourse of tissue localization, but does not consider the intensity, making it difficult to make quantitative conclusions on the inducibility of this gene by one bacteria over the other. Overall, the information presented is very disjointed; it is difficult to arrive at the authors’ conclustion that the expression of BaraA is strongly induced in a Toll-dependent manner in the fat body with the combination of the figures presented (1C & S1B)], but unresponsive to E. coli and the Imd pathway. The authors should be more thorough with their analysis or more judicious with their conclusions.

We appreciate the reviewer’s interest in the gene expression pattern. We will note that one should not conflate stimulus by a pathogen with regulation by a pathway, as these are correlated but distinct observations. For instance, the gram-negative bacterium Providencia rettgeri principally activates Imd signalling through its DAP-type peptidoglycan, but also activates Toll signalling via Persephone (Duneau et al., 2017; BMC Biology), likely by cleaving/activating Persephone with microbial proteases or triggering host danger signals (Issa et al., 2018; Mol Cell).

We also note that Relish is the terminal transcription factor of the Imd pathway, and so the comment that other components of Imd might contribute is covered by the fact that Imd signalling converges at the point of Relish activation – i.e. Relish is the terminal end of the Imd pathway. Thus, we weigh the genetic evidence using Relish and spz mutants in Fig1A-B and FigS1B over pathogen use in our interpretation. However we have also shored up these data with a new set of experiments in wild-type flies to better illustrate the nature of AMP expression (point-by-point below).

i) The methodology of figure 1C, which indicates a more robust response to M. luteus (but not unresponsive to E. coli - the use of “not” in the text and figure legend is unjustified) is not explained in either the text or the legend.

We have modified the text in Line 144 and the Fig. 1C caption to say “less so by E. coli” rather than “not by E. coli.”

ii) Further, the analysis in figure 1C appears to be only a binary call, based on a reporter over a timecourse of tissue localization, but does not consider the intensity, making it difficult to make quantitative conclusions on the inducibility of this gene by one bacteria over the other.

The quantification in Fig. 1C is intrinsically tied to intensity, as the systemic BaraA response is derived from the cumulative intensity of the mounting whole-body transcriptional response. We could have alternately written “unchallenged, low GFP intensity, high GFP intensity” and the resulting analysis would be the same.

iii) The authors claim BaraA is highly induced in the fat body by M. luteus but not E. coli, and triggered by the Toll pathway and not the Imd pathway, but S1B showed BaraA being induced strongly (45x) at 6hpi by E. coli, and lesser extent (6x) by M. luteus at 24hpi.

We have now produced a replicate set of experiments where we infected wild-type flies with E. coli or M. luteus and collected pooled samples 24hpi. This set of replicate experiments shows a clear resolution of BaraA back to nearly basal levels after E. coli pricking 24hpi, agreeing with our reporter data and another study that specifically comments on the BaraA expression pattern after Imd stimulus (Schlamp et al., 2021; BMC Genomics).

To address the comment on extent of induction: this second set of experiments (new Fig. S1C) shows BaraA induction of ~30x after M. luteus (similar to levels after C. albicans infection in Fig1B). Such is the fickle nature of AMP induction, where even the canonical Imd readout Diptericin is induced anywhere from 100x-5000x depending on study. One major factor that contributes to this stochasticity in AMP gene expression is the status of the unchallenged reference point. Even a mild elevated background expression of the AMP in the “unchallenged” flies greatly reduces the apparent induction in the infected state by virtue of shifting the baseline. There is also some variation in the induced state, and so these two factors combined can cause large fluctuations in the normalized induction.

iv) The first four residues of the C-terminal in D. melanogaster BaraA were not mentioned in the text. They were identified as GIND motif in the figure 1B legend, different from a furin cleavage motif (RXRR) at this site in other Drosophila species. The figure 2B itself, however, did not make this distinction clear and denoted this site as a furin cleavage site similar to other sites in D. melanogaster. Is this GIND motif a cleavage site for furin as well? Or is it cleaved by something else? Is there a significance in this site being different in D. melanogaster vs other Drosophila species?

We have revised Figure S3 to show that there is a furin cleavage site upstream* of GIND to avoid confusion; our previous figure was confusing on this point in retrospect admittedly.

The significance is that IM22 has a relatively fixed N-terminus, even when different cleavage mechanisms are used to maturate the peptide to that point. This provides insight that the IM22 peptide N-terminus is not easily tinkered with, implying the molecule is selected to maintain this exact protein length/structure.

**Part III – Minor Issues: Editorial and Data Presentation Modifications**
Please use this section for editorial suggestions as well as relatively minor modifications of existing data that would enhance clarity.

Reviewer #1: I found two typos or spelling mistakes:
In the abstract (line 23) Drososphila melanogaster -> Drosophila melanogaster
Line 134: ...flies similar tolike the Toll-regulated BomBc3 -> ...flies similar to the Toll-regulated BomBc3

Much appreciated for catching these typos.

Reviewer #2: (No Response)

Reviewer #3: Does the duplication event affect expression? It would be nice to have a brief comment about this in this paper regardless of the content of the other preprint.

Seemingly yes, though we have not done experiments with a controlled genetic background yet and prefer not to comment in the manuscript without this verification first. We note the duplication most importantly because it is relevant to our mutant generation method (choosing a recipient genotype with only one copy, while the reference genome has two copies). Of note, we do in fact present data in Fig. S10 that may be of interest.

Briefly, OR-R flies with the duplication seem to reach a higher induced level of expression than a standard w1118 with only one gene copy. The raw CT value data in Fig. S9E also show this. However this is complex to introduce into the early part of the present manuscript, and again we would prefer to verify this using a controlled genetic background before making resolute claims.

Thus while we report data that can be used to infer this trend, our intent for this data is just to inform our interpretations on expression in the transheterozygote state etc… and we prefer to not comment on the duplication and expression until we can repeat these findings with isogenic flies.

The diagram depicting the deletion in figure 2A is hard to read. It is too small and contains too little detail. It also looks as if the authors were trying to line the deletion diagram up with the MALDI-TOF graph, but these two graphs are not related to one another.

We have increased its relative size to make it easier to read.
